# Functional cardiotoxicity assessment of cosmetic compounds using human-induced pluripotent stem cell-derived cardiomyocytes

**DOI:** 10.1007/s00204-017-2065-z

**Published:** 2017-09-22

**Authors:** Umesh Chaudhari, Harshal Nemade, Poornima Sureshkumar, Mathieu Vinken, Gamze Ates, Vera Rogiers, Jürgen Hescheler, Jan Georg Hengstler, Agapios Sachinidis

**Affiliations:** 1 Institute of Neurophysiology and Center for Molecular Medicine Cologne (CMMC), University of Cologne (UKK), Robert-Koch-Str. 39, 50931 Cologne, Germany; 2 Department of In Vitro Toxicology and Dermato-Cosmetology, Faculty of Medicine and Pharmacy, Vrije Universiteit Brussel, Laarbeeklaan 103, 1090 Brussels, Belgium; 3 Leibniz Research Centre for Working Environment and Human Factors, Technical University of Dortmund (IfADo), 44139 Dortmund, Germany

**Keywords:** Cosmetics, Non-animal testing, Cardiotoxicity, Safety assessment, Human pluripotent stem cells, Cardiomyocytes

## Abstract

There is a large demand of a human relevant in vitro test system suitable for assessing the cardiotoxic potential of cosmetic ingredients and other chemicals. Using human-induced pluripotent stem cell-derived cardiomyocytes (hiPSC-CMs), we have already established an in vitro cardiotoxicity assay and identified genomic biomarkers of anthracycline-induced cardiotoxicity in our previous work. Here, five cosmetic ingredients were studied by the new hiPSC-CMs test; kojic acid (KJA), triclosan (TS), triclocarban (TCC), 2,7-naphthalenediol (NPT), and basic red 51 (BR51) based on cytotoxicity as well as ATP assays, beating rate, and genomic biomarkers to determine the lowest observed effect concentration (LOEC) and no observed effect concentration (NOEC). The LOEC for beating rate were 400, 10, 3, >400, and 3 µM for KJA, TS, TCC, NPT, and BR51, respectively. The corresponding concentrations for cytotoxicity or ATP depletion were similar, with the exception of TS and TCC, where the cardiomyocyte-beating assay showed positive results at non-cytotoxic concentrations. Functional analysis also showed that the individual compounds caused different effects on hiPSC-CMs. While exposure to KJA, TS, TCC, and BR51 induced significant arrhythmic beating, NPT slightly decreased cell viability, but did not influence beating. Gene expression studies showed that TS and NPT caused down-regulation of cytoskeletal and cardiac ion homeostasis genes. Moreover, TS and NPT deregulated genomic biomarkers known to be affected also by anthracyclines. The present study demonstrates that hiPSC-CMs can be used to determine LOECs and NOECs in vitro, which can be compared to human blood concentrations to determine margins of exposure. Our in vitro assay, which so far has been tested with several anthracyclines and cosmetics, still requires validation by larger numbers of positive and negative controls, before it can be recommended for routine analysis.

**Electronic supplementary material:**

The online version of this article (doi:10.1007/s00204-017-2065-z) contains supplementary material, which is available to authorized users.

## Introduction

Various kinds of cosmetic products overflow the market. They are used by billions of people of different ages, often on a daily basis. This is the case for general-use cosmetics such as cleansing cosmetics (shower gel, hand wash liquid, shampoo, and toothpaste), general skin care products (moisturizing day and night creams, body lotions, sun protection, and shaving aids), underarm deodorants, and perfumes. More specific products with active ingredients such as anti-aging creams, skin-bleaching products, and hair dyes have also become increasingly popular. Moreover, a whole market exists for baby cosmetics. As cosmetic products are in fact a mixture of chemicals of synthetic as well as natural origin, and human exposure is high, it is important that efficient safety measures are in place and followed-up on a regular basis.

Safety of cosmetics brought on the European market is of high priority in the current European Union (EU) cosmetic regulation 1223/2009/EC and is based on the safety of the ingredients (chemical structure, toxicological profile, and exposure). Special measures have been installed to deal with the occurrence of so-called serious unwanted effects (SUE). Although not commonly seen, serious side effects may occur. Indeed, cosmetic ingredients may reach the blood via absorption through the gastrointestinal tract, inhalation system, or the skin (Lin 2000; Lin et al. 1994; Sandborgh-Englund et al. 2006) and cause structural and functional toxicity to vital organs, such as the heart. Concern goes in particular to the unborn life, babies, and children.

It has been shown that embryonic exposure to cosmetic compounds can cause direct damage to the developing heart or interrupt heart development (Manjunatha et al. 2014). In the urban population of Brooklyn, New York, where exposure to triclosan (TS) and triclocarban (TCC) is high, it could be shown that pregnant women were more vulnerable to adverse health outcomes (Pycke et al. 2014). As these ingredients are usually discharged into local aquifers, deleterious effects on embryo development and heart function of aquatic species also cannot be excluded. TS-induced embryotoxicity and cardiotoxicity have been reported in zebrafish (Oliveira et al. 2009; Saley et al. 2016). Therefore, screening new cosmetic ingredients for cardiac safety seems warranted. This cannot be done anymore using experimental animals as the testing and marketing bans, present in the EU cosmetic regulation, fully apply from March 2013 onwards. This means that for regulatory purposes only validated replacement methods may be used. Following this ban, cosmetic industries urgently need human relevant in vitro models to test the safety of their products. Recent developments in stem cell technology made it possible to isolate specialized somatic cells of human origin. There is increasing evidence that human embryonic stem cells (hESCs) and human-induced pluripotent stem cells (hiPSCs), as well as their somatic cell derivatives, can be used in combination with transcriptomics for the development of suitable models to evaluate the potential toxicity of new chemical entities (Chaudhari et al. 2016b; Doss et al. 2012; Meganathan et al. 2012; Stummann et al. 2008; Waldmann et al. 2014). Recently, we established a functional test system for testing of potential cardiotoxicants using hiPSC-derived cardiomyocytes (hiPSC-CMs) and we also identified 35 genomic and several microRNA biomarkers of anthracyclines relevant to cardiotoxicity (Chaudhari et al. 2016a, b).

In this study using hiPSC-CMs, the effects of five cosmetic compounds on the cytotoxicity, cell viability, beating rate, beating pattern, and total intracellular adenosine triphosphate (ATP) content were evaluated. Based on our previous findings (Chaudhari et al. 2016b), we also investigated the mRNA expression of 84 genes (relevant to cardiotoxicity) to validate the specificity of the genomic biomarkers of anthracycline-induced cardiotoxicity. The cosmetic ingredients studied were divided into three classes, according to their function (Supplementary Table S1). Kojic acid (KJA) is an anti-oxidant used as a skin-whitening agent in various types of cosmetic products. KJA inhibits melanin synthesis (Kim et al. 2012). It has been approved in the EU at a concentration of 1%. Triclosan (TS) and Triclocarban (TCC) are lipid-soluble broad-spectrum anti-bacterial and anti-fungal agents commonly used in soaps, toothpastes, mouthwashes, shampoos, and cleansing products (Orsi et al. 2011). They have been approved in the EU in concentrations of 0.3 and 0.2%, respectively. Basic red 51 (BR51) and 2,7-naphthalenediol (NPT) are coloring agents for keratin fibers and are used in hair dyes (Eskelinen et al. 1997; Tafurt-Cardona et al. 2015). BR51 and NPT have been approved in the EU in concentrations of 1 and 0.68% (as free base), respectively.

## Materials and methods

### Cardiomyocyte cell cultures

**Fig. 1:**
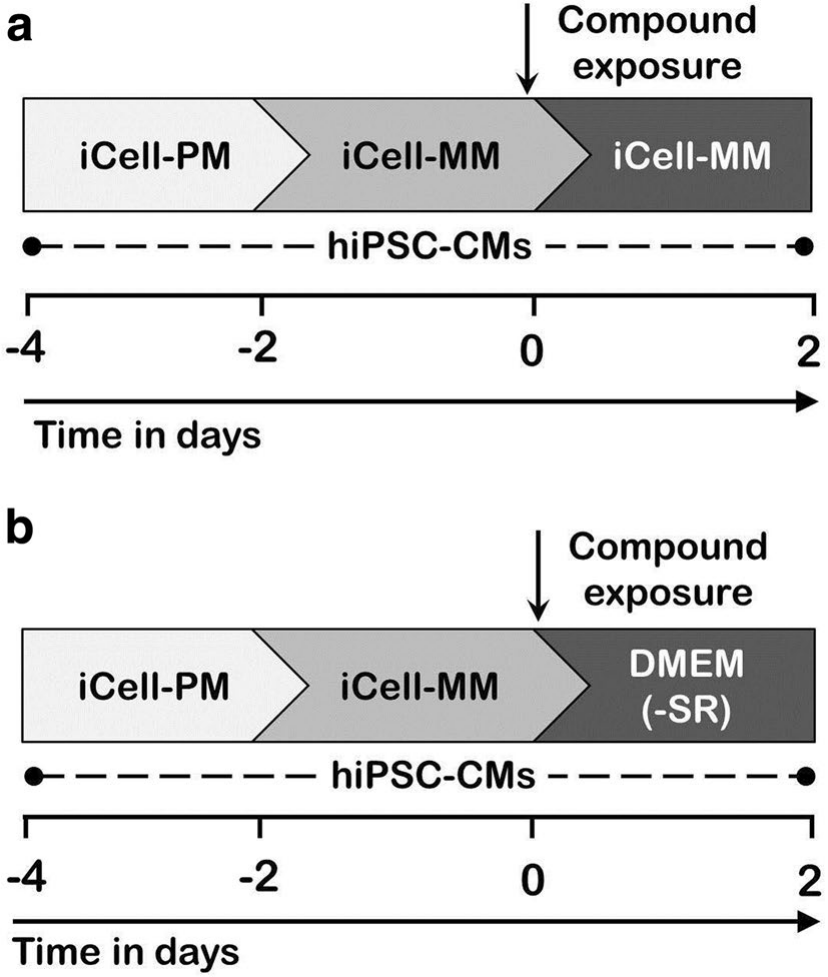
Schematic representation of experimental designs for in vitro safety assessment of cosmetic test compounds. **a** Experimental design to evaluate adverse effects of the cosmetic compounds in hiPSC-CMs under serum-containing culture medium (iCell-MM). **b** Experimental layout to study adverse effects of the cosmetic compounds in hiPSC-CMs in serum-free (−SR) culture medium; DMEM. Starting day of compound exposure was considered as day 0 in which hiPSC-CMs reached confluency. Following 2-day compound exposure, adverse effects of the compounds were analyzed using different readout assays

All experiments were conducted using highly purified (≥98%) and well-characterized non-proliferative human iCell Cardiomyocytes^®^ (Cellular Dynamics International, Madison, WI, USA). In this study, iCell cardiomyocytes, derived from hiPSCs, are referred to as hiPSC-CMs. The hiPSC-CMs were cultured, as described previously (Chaudhari et al. 2016b). In brief, cryopreserved hiPSC-CMs were thawed in a 37 °C water bath for 4 min, followed by slow addition of iCell cardiomyocyte plating medium (iCell-PM; Cellular Dynamics International) to the thawed hiPSC-CMs solution, as per the manufacturer’s instructions. These hiPSC-CMs were plated on fibronectin-coated (5 µg/cm^2^; 2 h at 37 °C) cell culture grade 6-well plates and 96-well assay plates. After a 2-day incubation, the iCell-PM was replaced with serum-containing iCell cardiomyocyte maintenance medium (iCell-MM; Cellular Dynamics International). 4 days after cell plating (day 0), hiPSC-CMs were exposed to cosmetic test compounds in serum or serum-free cell culture media (Fig. 1a, b). The hiPSC-CMs were cultured in a humidified air incubator, with 5% CO_2_ at 37 °C. For functional studies, approximately 25 × 10^3^ hiPSC-CMs were seeded per well of an E-plate Cardio 96 (ACEA Biosciences, San Diego, CA, USA) or a 96-well white assay plate. For gene expression studies, hiPSC-CMs were plated in 6-well plates, with a cell density of approximately 0.4 × 10^6^ per well.

### Cytotoxicity and cell viability assay

The hiPSC-CMs seeded in the 96-well white assay plates were exposed to different concentrations of the five cosmetic test compounds. After 2-day compound exposure, supernatant-culture media was used to measure the cytotoxic effect, while attached viable cells in the plate were used to determine cell viability. We used lactate dehydrogenase (LDH) leakage as an indicator of cell membrane damage. Cytotoxic effect of the five test compounds was evaluated by measuring the activity of extracellular LDH, leaked from the cardiomyocytes into the culture media in response to compound exposure. Culture medium from each well of the assay plate was transferred to a clear 96-well assay plate using a multi-channel pipette. A Thermo Scientific™ Pierce™ LDH Cytotoxicity Assay Kit was used to measure LDH activity, as per the manufacturer’s instructions. Absorbance was measured at 490 nm using a Softmax Pro M5e 96-well plate reader (Molecular Devices). Replicate samples of iCell-MM without cells were used as blanks. Inherent LDH activity in blank wells was used as a background signal. Cell viability was determined using a modified propidium iodide (PI) assay (Shirsath et al. 2013). Briefly, PI is a DNA intercalating agent and a fluorescent molecule. It is commonly used to evaluate cell viability or DNA content in cell cycle studies. After 2-day compound exposure, hiPSC-CMs were washed twice with phosphate buffer saline (PBS), followed by the addition of 200 µl of an aqueous PI solution (7 µg/ml) to each well. PI is impermeable to live cell membranes. To determine the proportion of viable cells, cells were permeabilized to PI by keeping the plate in the deep freezer (−20 °C) overnight. Freezing results in cell death, and makes cell membranes permeable to PI. After thawing of the plate, PI enters the cells and stains the nuclear DNA. Fluorescence from the resulting PI–nuclear DNA complex was measured using a Softmax Pro M5e 96-well plate reader (excitation *λ* 530 nm; emission *λ* 620 nm). Replicates of PI solution without cells were used as blank wells. Fluorescence values obtained from blank wells were considered to be the background fluorescence. Thus, fluorescence values collected from PI-stained cells are directly proportional to the number of viable cells.

### Cardiomyocyte impedance measurements

An impedance assay was performed using xCELLigence RTCA Cardio system (ACEA Biosciences). This system registers electrical impedance changes across synchronously beating cardiac monolayers and displays these data by converting impedance values into Cell Index (CI) values. The CI value (arbitrary unit) is directly proportional to the surface area covered by the attached cells in the E-plate Cardio 96. This system monitors change in cell morphology, cell viability, beating rate, and beating pattern in real time. Before cell seeding, background impedance readings were measured after the addition of 50 µl of iCell-PM to each well of an E-plate Cardio 96. The hiPSC-CMs plated on the E-plate Cardio 96 underwent a 2-day exposure to five test compounds at various concentrations. Impedance signals from each well of the E-plate Cardio 96 were collected at regular intervals. Cytotoxicity and beating data were analyzed using the RTCA Cardio software version 1.0 (ACEA Biosciences). To evaluate the effects of the five cosmetic test compounds on cell viability and cardiac beating function under a serum-free culture conditions, hiPSC-CMs underwent a 2-day exposure to these five test compounds in serum-free basal culture medium; ‘Dulbeccos’s Modified Eagle Medium’ (DMEM, Invitrogen, Darmstadt, Germany). In control cultures, dimethyl sulfoxide (DMSO) was used as the compound-free solvent.

### Measurement of total intracellular adenosine triphosphate levels in human-induced pluripotent stem cell-derived cardiomyocytes

The hiPSC-CMs plated in the 96-well white assay plate were exposed to various concentrations of the five test compounds. After 2-day exposure, total intracellular adenosine triphosphate (ATP) level was measured using ATPlite Luminescence ATP Detection Assay System kit (PerkinElmer, Netherlands), according to the manufacturer’s instructions. Briefly, viable cells were lysed by incubation with a mammalian cell lysis solution for 5 min on an orbital shaker. After cell lysis, the substrate solution was immediately added to all wells, and the assay plate was shaken for another 5 min. Following a 10 min incubation in the dark, luminescence levels were measured on a Softmax Pro M5e 96-well plate reader (Molecular Devices). Luminescence values are directly proportional to the amount of ATP.

### RNA extraction

The hiPSC-CMs plated in the 6-well plate underwent 2-day exposure to five test compounds (in the presense of serum) at three different concentrations, having none to slight cytotoxic effects. The hiPSC-CMs exposed to DMSO solvent and the test compounds were lysed and harvested using Qiazol cell lysis reagent (Qiagen, Hilden, Germany). Total RNA was extracted from lysed cell samples using miRNeasy Mini Kit (Qiagen), as per the manufacturer’s instructions. Concentration and purity of the extracted RNA were checked using a Nanodrop ND-1000 spectrophotometer (ND-1000, Thermo Fisher, Langenselbold, Germany). These extracted pure RNA samples were used for gene expression studies.

### Quantitative real-time polymerase chain reaction

Quantitative real-time polymerase chain reactions (qPCRs) were carried out, as described in our previous work (Chaudhari et al. 2016b). Briefly, 500 ng of total RNA was reverse transcribed into cDNA, using an RT^2^ First Strand Kit (Qiagen), according to the manufacturer’s instructions. These cDNA samples underwent quantitative amplification of target genes using a custom made RT^2^ profiler PCR array (96-well plate) (Qiagen) and RT^2^ SYBR Green ROX qPCR master mix (Qiagen). The RT^2^ profiler PCR array contained 84 target genes, 5 housekeeping genes, 1 genomic DNA control, 3 reverse transcription controls, and 3 positive PCR controls. The qPCR was performed using a 7500 FAST Real-Time PCR System (Applied Biosystems, Foster City, CA, USA), under the manufacturer’s recommended thermal cycling conditions. The qPCR was performed in triplicate for each sample. Gene expression data were normalized using the geometric mean of the five housekeeping genes, *ACTB, B2M, GAPDH, HPRT1,* and *RPLP0*. Control sample values were used as reference values. Relative changes in gene expression were calculated using the 2^−ΔΔCt^ method (Dardousis et al. 2007).

### Test compounds

The compounds KJA, TS, TCC, and NPT were purchased from Sigma-Aldrich (Darmstadt, Germany), while BR51 was purchased from Santa Cruz Biotechnology (Dallas, Texas, USA). The 40 mM stock solutions of all five cosmetic test compounds were prepared in DMSO solvent. Stock solutions were dispensed in small volume aliquots and maintained at −20 °C in storage. Compound dilutions were made in iCell-MM or serum-free basal medium DMEM just before compound exposure.

### Statistical data analysis

Raw data from ATP, LDH, and PI assays were exported to a text file, while Ct values from the qPCR studies and impedance data were exported to an Excel file. Analysis of qPCR data was performed using Qiagen’s RT^2^ PCR array data online analysis tool. Exported data from all readouts were analyzed, tabulated, and graphed in Excel 2010 (Microsoft, Redmond, WA, USA). Error bars in the figures represent mean ± standard deviation (SD). The statistical significance of the difference between the control and test compound-exposed group was determined using Student’s *t* test (Excel 2010), with *p* values < 0.05 considered to be significant.

## Results

### Cytotoxicity effects of the different compounds in the human-induced pluripotent stem cell-derived cardiomyocytes in the presence and absence of serum

The cytotoxic effect of each of the five cosmetic test compounds was evaluated using four different methods: (1) impedance measurements in the presence and absence of serum; (2) extracellular LDH activity (in the presence of serum); (3) fluorescence of propidium iodide (PI) stained hiPSC-CMs nuclei (in the presence of serum), and (4) by the quantification of total intracellular ATP content (in the presence of serum).

**Fig. 2:**
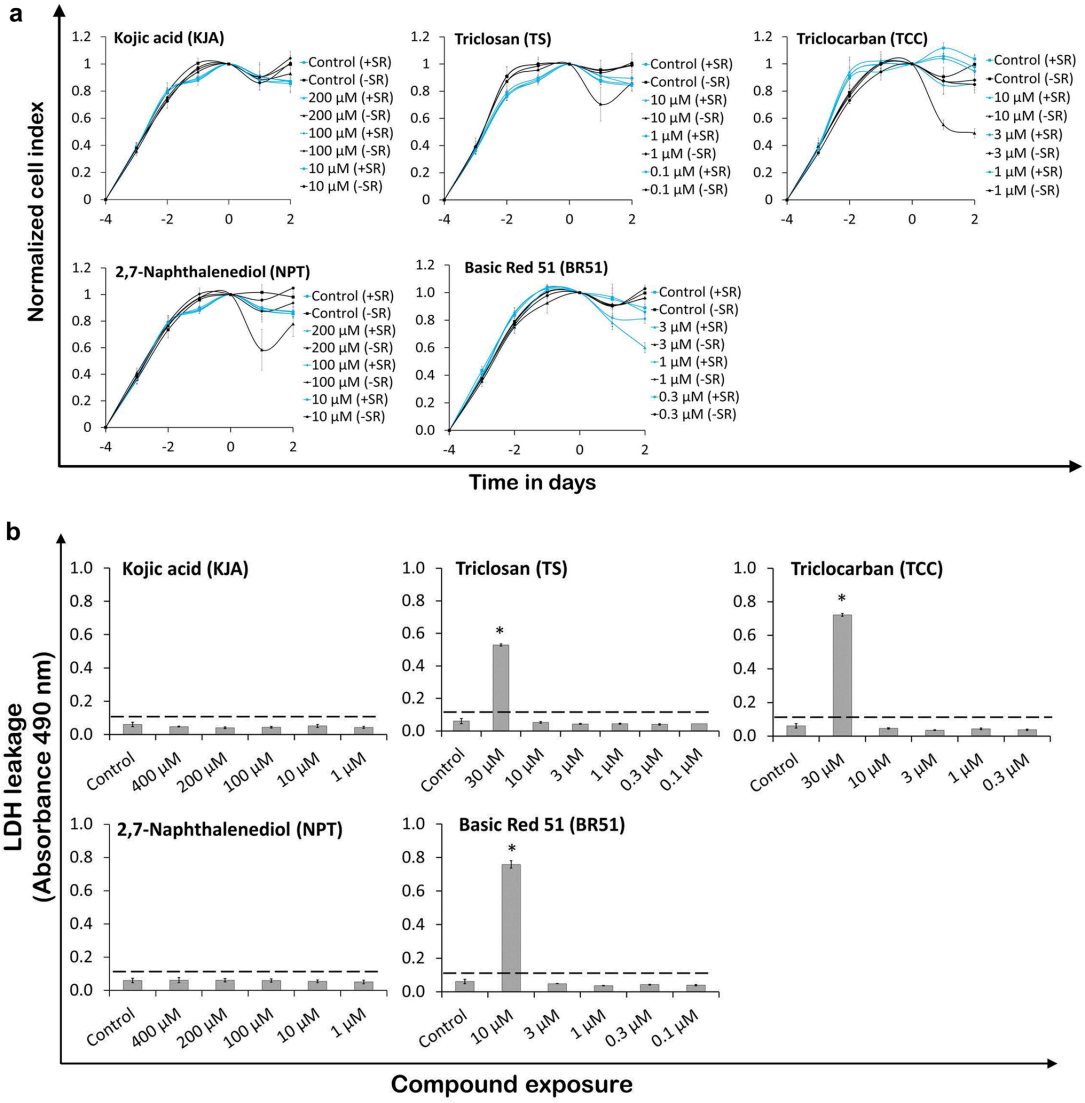

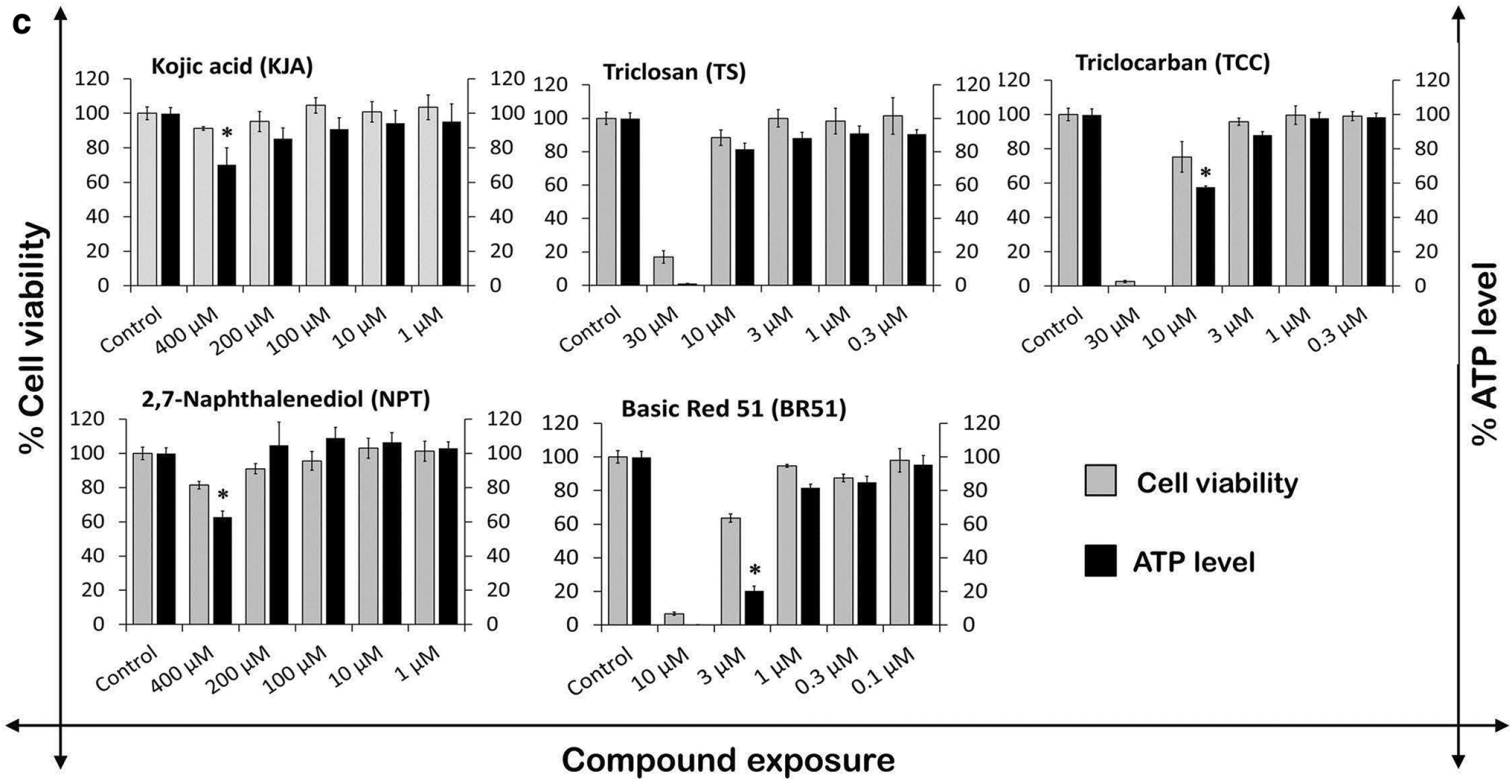
Evaluation of toxic effects of cosmetic test compounds on hiPSC-CMs. **a** Cytotoxicity assessment of test compounds in hiPSC-CMs under serum or serum-free culture conditions using impedance measurements applying the xCELLigence system. CI values were normalized at day 0 prior to compound exposure (mean ± SD, *n* = 3). **b** Effect of cosmetic compounds on LDH leakage in hiPSC-CMs in serum-containing culture medium. Activity of released LDH in cell culture medium by  DMSO solvent and compound-exposed cells was determined by absorbance readings (mean ± SD, *n* = 3, *p < 0.05 for the compound exposed vs control hiPSC-CMs). Dotted line represents basal LDH activity in blank wells. **c** Effect of cosmetic compounds on cell viability and total ATP content in hiPSC-CMs cultured in serum condition. Following 2-day compound exposure, cell viability data was collected using fluorescence values, while intracellular ATP levels were determined using luminometric values (mean ± SD, *n* = 3, *p < 0.05 for the compound exposed vs control hiPSC-CMs)

To evaluate cytotoxicity under serum-free culture conditions, three concentrations of each of the five cosmetic compounds were used. Impedance data showed that TS (10 µM) and NPT (200 µM) markedly lowered CI values after 1-day exposures, but after a further 1-day incubation, an elevation in CI values was observed (Fig. 2a). However, these elevated CI values did not reach the levels of controls. Compared to serum-containing culture conditions, this data suggests that TS (10 µM) and NPT (200 µM) induced morphological changes that occurred during early exposure, producing minor cytotoxic effects overall. In contrast to serum-containing culturing conditions, TCC at 10 µM markedly lowered CI values, indicating a cytotoxic effect. KJA and BR51 did not alter cell viability. Notably, the CI values of control hiPSC-CMs were not affected by the presence or absence of serum in their culturing media (Fig. 2a). In addition, higher concentrations have been tested in the presence of serum (KJA, 300 and 400 µM; TS, 30 µM; TCC, 30 µM; NPT, 400 and 300 µM; BR51, 10 µM). As shown in Supplementary Fig. S1, TCC at 30 µM as well as BR51 at 10 µM were extremely toxic even after 1 day of incubation, whereas higher concentrations of the other compounds were non-toxic (KJA) or slightly toxic (TS and NPT).

As shown in Fig. 2b, c, only the highest concentration of 30 µM TS, 30 µM TCC, and 10 µM BR51 increased extracellular LDH activity in the culture media, and reduced cell viability (measurements have been performed in the presence of serum). These results demonstrate that test compounds at high concentrations caused cytotoxicity and cell membrane damage, which resulted in a decline in cell viability. In contrast to impedance measurements and LDH leakage assays, TCC at 10 µM showed a sub-cytotoxic effect in the PI assay. NPT at a very high concentration of 400 µM had lower CI values (Supplementary Fig. S1), slightly decreased cell viability (Fig. 2c), but did not show extracellular LDH activity in the culture media (Fig. 2b). These observations suggest that NPT at high concentrations induced morphological changes in the cells, having a low cytotoxic effect that did not cause major damage to cell membrane integrity. The hiPSC-CMs exposed to KJA did not show changes in CI values (Supplementary Fig. S1), extracellular LDH leakage, or cell viability (Fig. 2b, c). Quantification of total intracellular ATP indicates the presence of metabolically active cells. The ATP data show that KJA (400 µM), TCC (10 µM), NPT (400 µM), and BR51 (3 µM) significantly decreased ATP levels (Fig. 2c), whereas lower concentrations did not show significant effects. In particular, TS at 10 µM decreased total ATP levels by 18% in hiPSC-CMs.

### Effects of the different compounds on beating activity of human-induced pluripotent stem cell-derived cardiomyocytes in the presence and absence of serum

Beating activity data in the presence of serum showed that KJA (400 µM), TS (30 and 10 µM), and TCC (10 µM) induced an increase of the beating activity in hiPSC-CMs (Supplementary Fig. S2). Lower concentrations did not show any influence. All test concentrations of NPT did not disturb the beating activity in hiPSC-CMs. In contrast, BR51, even at 3 µM sub-cytotoxic concentrations, caused arrhythmic beating of hiPSC-CMs, resulting in a lower beating rate (Supplementary Fig. S2); however, concentrations below 3 µM did not have any effect.

**Fig. 3:**
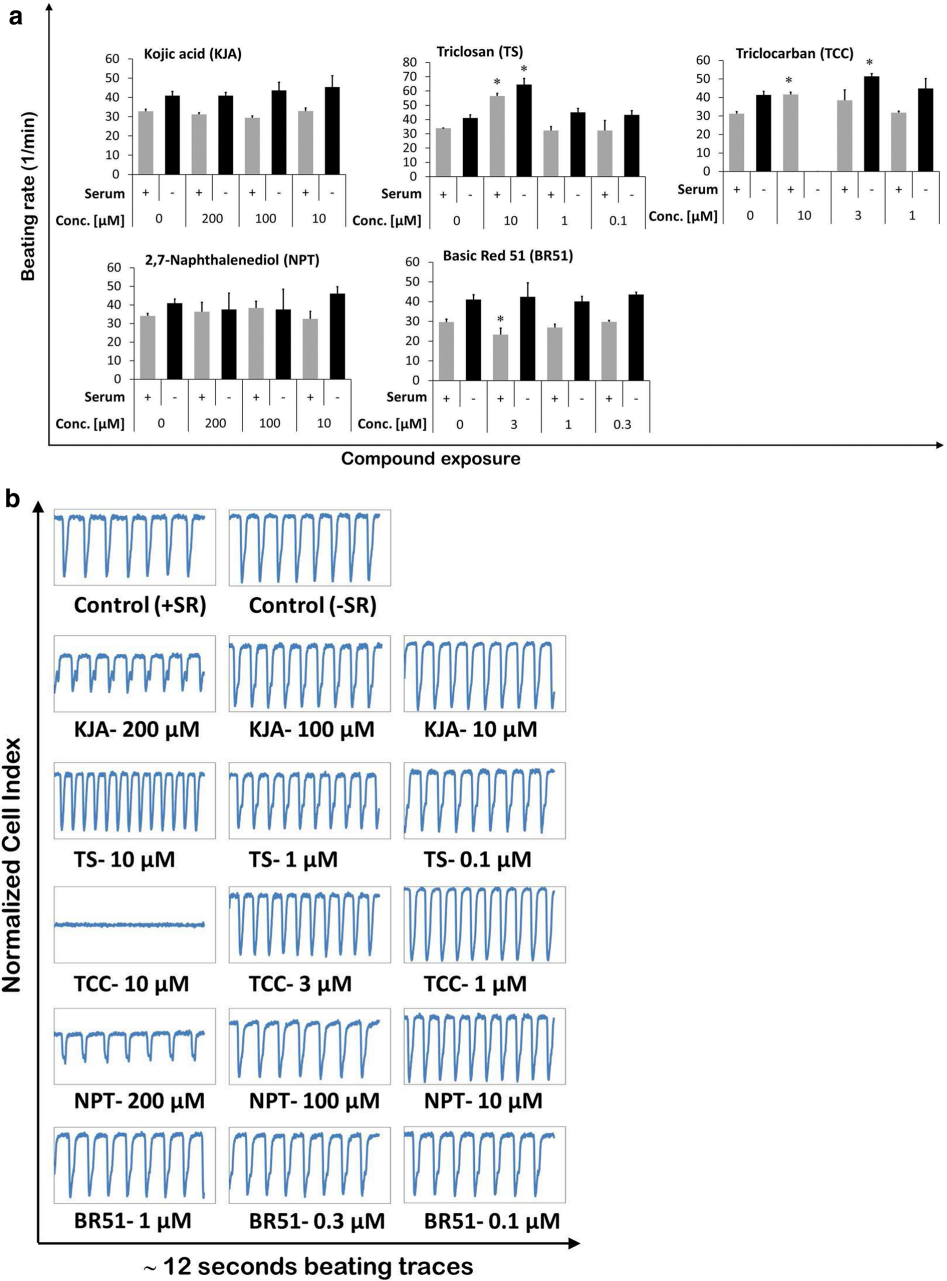
Evaluation of effect of cosmetic test compounds on beating function of hiPSC-CMs. **a** Beating rate in hiPSC-CMs was analyzed after 2 days of exposure to DMSO solvent and cosmetic compounds under serum and serum-free culture conditions. Beating rate was determined using the xCELLigence RTCA Cardio software version 1.0 at threshold 12 (mean ± SD, *n* = 3, *p < 0.05 for compound exposed vs control hiPSC-CMs). **b** Representative 12 s beating traces of cardiomyocytes after 2 days of exposure to DMSO solvent and cosmetic test compounds under serum-free culture condition. *Control* (*+SR*) hiPSC-CMs exposed to DMSO in serum-containing culture medium, *Control* (*−SR*) hiPSC-CMs exposed to DMSO in serum-free culture medium, *KJA* kojic acid, *TS* triclosan, *TCC* triclocarban, *NPT* 2,7-naphthalenediol, and *BR51* basic red 51

Similar to the results obtained with serum-containing culture medium, TS (10 µM) significantly increased the beating rate of the hiPSC-CMs also under serum-free conditions (Fig. 3a) and induced an arrhythmic beating pattern (Fig. 3b). Unlike serum-containing culturing conditions, TCC (10 µM) completely suppressed beating in the hiPSC-CMs (Fig. 3a, b). In addition, TCC at 3 µM significantly increased the beating rate in hiPSC-CMs. Similar to serum-containing culturing conditions (Supplementary Fig. S2), KJA and NPT did not change the beating rate. However, at 200 µM, they considerably reduced beating peak heights in hiPSC-CMs (Fig. 3b). BR51 did not show any adverse effects on beating rate in hiPSC-CMs. Moreover, the beating data showed that the control hiPSC-CMs cultured in serum-free conditions showed a marginal increase in beating rate, compared to those cultured under serum-containing conditions.

### Effects of the test compounds on expression of genes involved in cardiac functions

**Fig. 4:**
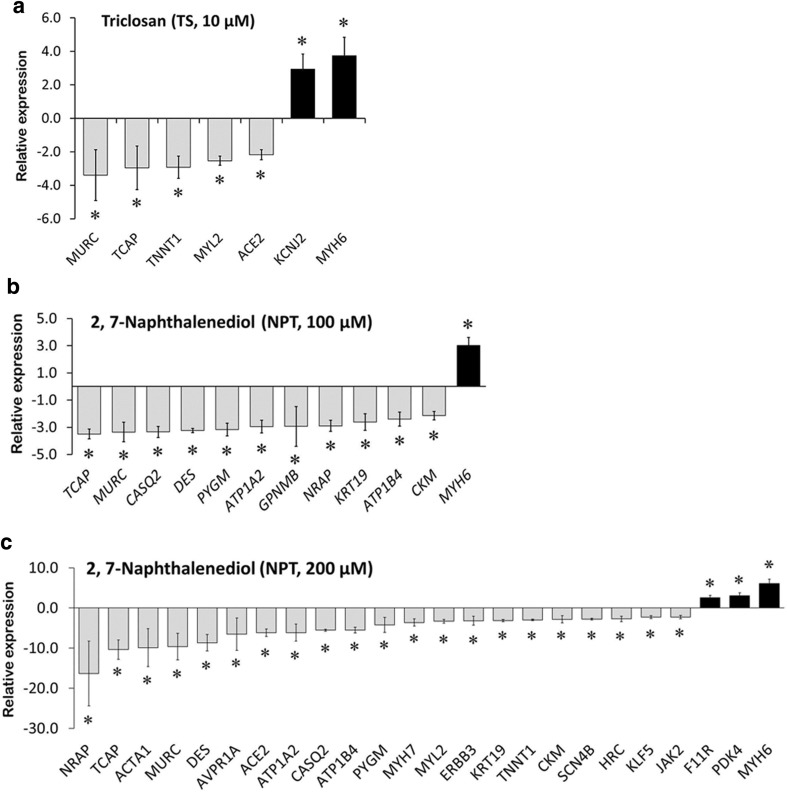
Relative gene expression studies using qPCR. Significant differential expression of genes by TS at 10 µM (**a**), NPT at 100 µM (**b**), and NPT at 200 µM (**c**). Bar graph shows the fold change differences in expression levels of target genes between control and compound-exposed cardiomyocytes (mean ± SD, *n* = 3, *p < 0.05, fold change ≥2.0)

To evaluate the influence of KJA, NPT, and TS on gene expression, three concentrations ranging from non-toxic to marginally cytotoxic were investigated in the presence of serum. The influence on 84 target genes was studied using qPCR. These genes were chosen, because they are differentially expressed after exposure to the cardiotoxic cytostatic drug doxorubicin (156 nM), an anthracycline compound in genome-wide expression studies (Supplementary Table S2). Moreover, two further anthracycline compounds—daunorubicin and mitoxantrone, commonly deregulated 35 of these 84 genes. These 35 genes have recently been proposed as genomic biomarkers of anthracycline-induced cardiotoxicity (Supplementary Table S3) (Chaudhari et al. 2016b). To quantify the influence of the test compounds on gene expression, a “Cardiotoxicity Index” (CTI_84g_) was defined. This index has a maximal value of 1, which is reached when all 84 analyzed genes are deregulated by a compound; likewise, it has a minimal value of 0 if no gene is deregulated. TS (10 µM) deregulated the expression of 7 cardiotoxicity genes (5 down- and 2 up-regulated), indicating CTI_84g_ of 0.08 (Fig. 4a). NPT at concentrations of 100 and 200 µM significantly down-regulated the expression of 11 and 21 genes, while up-regulating the expression of 1 and 3 genes, respectively (Fig. 4b, c). This yields CTI_84g_ values of 0.14 and 0.28 for 100 and 200 µM NPT, respectively. At 10 µM, TS-induced up-regulation of two genes, related to cardiac contraction; the NPT-induced up-regulated genes were related to cardiac function and energy metabolism. No significant alteration in mRNA expression of the 84 target genes was observed in hiPSC-CMs exposed to different concentrations of KJA (CTI_84g_ value = 0) (data not shown). Likewise, TS (at 0.1 and 1 µM) and NPT (at 10 µM) did not deregulate any target genes (CTI_84g_ value = 0).

## Discussion

In principle, several cosmetic compounds may cause cardiac dysfunction and even heart failure (Feenstra et al. 1999; Pai and Nahata 2000). However, human blood concentrations after exposure to cosmetics are usually low and may be below critical threshold concentrations. Since in vivo testing of cosmetics is no longer possible, the establishment of in vitro assays to predict cardiotoxic effects of cosmetic ingredients and critical concentration ranges is of high relevance.

Functional analysis of hiPSC-CMs showed that KJA, TS, TCC, and BR51 can influence the beating rate (Fig. 3a). In other studies, Japanese rice fish (*Oryzias latipes*) embryos exposed to TS responded with a significant increase in heart-beating rate (Nassef et al. 2010). TS also caused cardiac dysfunction in the left ventricular cardiomyocytes of the mouse heart (Cherednichenko et al. 2012). In contrast, no effects of TS on zebrafish heart-beating rate were observed (Oliveira et al. 2009; Shim et al. 2016). Therefore, in case of TS, zebrafish may not be a suitable model to extrapolate to human cardiotoxicity. Our data indicate that NPT within the range of tested concentrations had no effect on the beating rate of cardiomyocytes (Fig. 3a). Moreover, none of the cosmetic compounds induced arrhythmic conditions in hiPSC-CMs after a 48 h washout period (Supplementary Fig. S3). Disruption in ATP production may result in cardiac contractile dysfunction or even heart failure (Gaspar et al. 2014). Our findings showed that KJA, TCC, NPT, and BR51 reduced ATP production in hiPSC-CMs. TS has been reported to cause mitochondrial uncoupling in zebrafish, rat, and human mast cells, as well as in primary human keratinocytes (Shim et al. 2016; Weatherly et al. 2016). Consistent with these data, our study showed 18% reduction in total ATP linked to TS, although this effect did not amount to statistical significance (Fig. 2c).

Many compounds bind to serum proteins like albumin. Therefore, free drug concentrations might be lowered in the blood (in vivo situation) or in culture medium containing serum. Therefore, in this study, non-toxic to low cytotoxic effects could be obtained because of a reduction in freely available test compounds in the medium. To rule out this possibility, we evaluated the cardiotoxic effects of the five cosmetic compounds at three different concentrations under serum-free culture conditions. Our findings suggest that in serum-free conditions, cardiomyocytes exposed to TS (10 µM), TCC (10 and 3 µM) and NPT (200 µM) experienced structural damage and/or cardiac contractile dysfunction. Moreover, serum-free test concentrations of BR51 (up to 2 µM) can be considered as safe for cardiac health, since even at high concentrations, no impacts were recorded on cell viability or beating function. Arrhythmic beating of hiPSC-CMs during test compound incubation under serum-free culturing conditions also recovered to basal values after a 48 h washout period of the compounds (Supplementary Fig. S3).

Data obtained from the here established in vitro cardiotoxicity assay can be used for an approximate assessment of human risk if human biomonitoring data are available. For TS, numerous human studies have been performed to determine blood concentrations. For example, it has been reported total baseline plasma levels of healthy volunteers ranging between 0.35 and 28 nM (0.1–8.1 µg/l) (Sandborgh-Englund et al. 2006). In another study, mean TS concentrations of 2.2 nM (0.648 µg/l) were obtained in maternal blood (Wei et al. 2017). An age-dependent analysis of TS in blood serum resulted in the highest average in 31–45-year-old males with 66 nM (19 ng/g) (Allmyr et al. 2008). A relatively extreme but still not unrealistic scenario was studied by Sandborgh-Englund (2006) who determined human pharmacokinetics of an oral dose of 4 mg TS. Such an exposure would correspond to a scenario, where an individual swallows approximately 1.3 g of a toothpaste that contains 0.3% TS, a scenario that may be considered as unusual but not impossible. This oral dose of 4 mg TS resulted in a mean plasma peak concentration of 753 nM (218 µg/l) in ten individuals. The individual with the highest plasma peak concentration reached 1.22 µM (354 µg/l) (Sandborgh-Englund et al. 2006). These human blood concentrations can be used for a comparison with lowest observed effect concentrations (LOECs) in the in vitro test obtained in the present study. The LOEC for cytotoxicity of TS was 30 µM, while 10 µM did not induce any cytotoxicity in hiPSC-CMs. Functional tests showed that the beating rate of hiPSC-CMs was already influenced at 10 µM TS, while 3 µM did not change the beating rate. At a concentration of 10 µM TS, a panel of cardiotoxicity associated genes showed altered expression levels. Therefore, the panel of in vitro tests performed in the present study led to a LOEC of 10 µM, while 3 µM can be considered as no observed effect concentration (NOEC). This NOEC of 3 µM is clearly higher than baseline concentrations of TS in human blood that have been reported to range between 0.35 and 66 nM (Allmyr et al. 2008; Sandborgh-Englund et al. 2006; Wei et al. 2017). In addition, the ‘worst case scenario’ of swallowing 1.3 g of toothpaste with 0.3% TS would still result in lower plasma peak concentrations compared the NOEC in vitro. However, the margin of exposure with a factor of less than 3 comparing the plasma peak concentration of 1.22 µM and the NOEC of 3 µM is relatively small and illustrates that further studies are warranted to obtain more comprehensive insight of human risk of cardiotoxicity due to TS exposure. However, it should be considered that the here presented in vitro test with hiPSC-CMs still has to be interpreted with caution. Comprehensive validation studies with positive and negative controls still have to be finished. Therefore, it is presently unknown how precisely, the here studied cardiomyocytes in vitro represent the susceptibility of real cardiomyocytes in a human heart. Moreover, the strategy of comparing total plasma peak blood concentrations in vivo to permanent concentrations of the test compound in the culture medium in vitro may also be associated with uncertainties that still have to be understood in detail.

A similar evaluation as described above for TS can also be performed for TCC. In a study of maternal serum, mean concentrations of 0.94 nM (0.292 µg/l) have been reported (Wei et al. 2017). The here performed in vitro test with hiPSC-CMs resulted in LOEC of 3 µM for beating activity, while 1 µM (NOEC) led to negative test results. Compared to the human blood concentrations reported by Wei et al. (2017), the margin of exposure is more than 1000-fold. However, it still should be discussed with caution, whether human ‘worse case exposure scenarios’ for TCC may not result in higher human blood concentration. To our knowledge, studies that aim to estimate the highest possible human blood concentrations of TCC under high but still not unrealistic exposure scenarios are still not available.

For KJA, a study is available, where a mean *C*_max_ in blood of 1.54 ng/ml, corresponding to approximately 10.8 nM, have been reported in healthy Japanese women, after a single application of a facial cream containing 1% KJA (Burnett et al. 2010). The only positive in vitro effect of KJA was obtained for the beating rate at 400 µM. Therefore, the margin of exposure is approximately 40,000-fold. In contrast to TS, TCC, and KJA, information on human blood concentrations is difficult to obtain for NPT and BR51, which limits the possibilities of human risk evaluation.

Moreover, non-toxic concentrations of the five cosmetic ingredients explored in this study can be considered as safe to human and animal hearts when such concentrations have CTI_84g_ values of close to zero. The CTI_84g_ value is a very sensitive cytotoxicity parameter, which is based on the deregulation of 84 genes in cardiomyocytes induced by a severe gold standard cardiotoxicant such as doxorubicin. The CTI_84g_ value covers functional and metabolism genes essential for an intact cardiomyocyte function.

In summary, we demonstrated that hiPSC-CMs can in principle be applied for safety assessment of cosmetics by comparing human total blood concentrations under relevant exposure scenarios to NOECs in vitro, which allows determination of a margin of exposure. The major challenge in the future will be to validate the hiPSC-CMs in vitro test by analysis of a larger number of well-established cardiotoxic compounds and negative controls with known human blood concentrations.

## Electronic supplementary material

Below is the link to the electronic supplementary material.

**Supplementary Fig. S1** Impedance-based cytotoxicity assessment of cosmetic test compounds in hiPSC-CMs in the presence of serum. Cytotoxic effect of test compounds in hiPSC-CMs was determined after 2 days of exposure using the xCELLigence RTCA Cardio software version 1.0. CI were normalized on day 0 just before start of compound exposure. Data represent mean ± SD, n = 3. **Supplementary Fig. S2** Determination of arrhythmogenic effect of cosmetic test compounds on the beating rate of hiPSC-CMs in the presence of serum. Beating rate data were collected after 2 days of test compound exposure using the xCELLigence RTCA Cardio software version 1.0 at threshold 12. Numerical data presented as mean ± SD (n = 3), *p < 0.05 for the compound exposed vs control hiPSC-CMs. **Supplementary Fig. S3** Evaluation of adverse effect of test compounds on the beating rate of hiPSC-CMs in the presence and absence of serum after 48 h compound washout. Beating rate data were obtained from the xCELLigence RTCA Cardio software version 1. In bar graph, error bar indicates mean ± SD (n = 3) (PDF 398 kb)

**Supplementary table S1.** Classification and applicability of the cosmetic ingredients in commercial cosmetic products (TIFF 875 kb)

Supplementary material 3 (XLSX 16 kb)

## References

[CR1] Allmyr M, Harden F, Toms LM, Mueller JF, McLachlan MS, Adolfsson-Erici M, Sandborgh-Englund G (2008). The influence of age and gender on triclosan concentrations in Australian human blood serum. Sci Total Environ.

[CR2] Burnett CL, Bergfeld WF, Belsito DV, Hill RA, Klaassen CD, Liebler DC, Marks JG, Shank RC, Slaga TJ, Snyder PW, Andersen FA (2010). Final report of the safety assessment of Kojic acid as used in cosmetics. Int J Toxicol.

[CR3] Chaudhari U, Nemade H, Gaspar JA, Hescheler J, Hengstler JG, Sachinidis A (2016). MicroRNAs as early toxicity signatures of doxorubicin in human-induced pluripotent stem cell-derived cardiomyocytes. Arch Toxicol.

[CR4] Chaudhari U, Nemade H, Wagh V, Gaspar JA, Ellis JK, Srinivasan SP, Spitkovski D, Nguemo F, Louisse J, Bremer S, Hescheler J, Keun HC, Hengstler JG, Sachinidis A (2016). Identification of genomic biomarkers for anthracycline-induced cardiotoxicity in human iPSC-derived cardiomyocytes: an in vitro repeated exposure toxicity approach for safety assessment. Arch Toxicol.

[CR5] Cherednichenko G, Zhang R, Bannister RA, Timofeyev V, Li N, Fritsch EB, Feng W, Barrientos GC, Schebb NH, Hammock BD, Beam KG, Chiamvimonvat N, Pessah IN (2012). Triclosan impairs excitation-contraction coupling and Ca^2+^ dynamics in striated muscle. Proc Natl Acad Sci USA.

[CR6] Dardousis K, Voolstra C, Roengvoraphoj M, Sekandarzad A, Mesghenna S, Winkler J, Ko Y, Hescheler J, Sachinidis A (2007). Identification of differentially expressed genes involved in the formation of multicellular tumor spheroids by HT-29 colon carcinoma cells. Mol Ther.

[CR7] Doss MX, Di Diego JM, Goodrow RJ, Wu Y, Cordeiro JM, Nesterenko VV, Barajas-Martinez H, Hu D, Urrutia J, Desai M, Treat JA, Sachinidis A, Antzelevitch C (2012). Maximum diastolic potential of human induced pluripotent stem cell-derived cardiomyocytes depends critically on I(Kr). PLoS One.

[CR8] Eskelinen A, Molitor C, Kanerva L (1997). Allergic contact dermatitis from 2,7-dihydroxynaphthalene in hair dye. Contact Dermat.

[CR9] Feenstra J, Grobbee DE, Remme WJ, Stricker BH (1999). Drug-induced heart failure. J Am Coll Cardiol.

[CR10] Gaspar JA, Doss MX, Hengstler JG, Cadenas C, Hescheler J, Sachinidis A (2014). Unique metabolic features of stem cells, cardiomyocytes, and their progenitors. Circ Res.

[CR11] Kim H, Choi HR, Kim DS, Park KC (2012). Topical hypopigmenting agents for pigmentary disorders and their mechanisms of action. Ann Dermatol.

[CR12] Lin YJ (2000). Buccal absorption of triclosan following topical mouthrinse application. Am J Dent.

[CR13] Lin YJ, Fung KK, Kong BM, DeSalva SJ (1994). Gingival absorption of triclosan following topical mouthrinse application. Am J Dent.

[CR14] Manjunatha B, Wei-bing P, Ke-chun L, Marigoudar SR, Xi-qiang C, Xi-min W, Xue W (2014). The effects of henna (hair dye) on the embryonic development of zebrafish (*Danio rerio*). Environ Sci Pollut Res Int.

[CR15] Meganathan K, Jagtap S, Wagh V, Winkler J, Gaspar JA, Hildebrand D, Trusch M, Lehmann K, Hescheler J, Schluter H, Sachinidis A (2012). Identification of thalidomide-specific transcriptomics and proteomics signatures during differentiation of human embryonic stem cells. PLoS One.

[CR16] Nassef M, Kim SG, Seki M, Kang IJ, Hano T, Shimasaki Y, Oshima Y (2010). In ovo nanoinjection of triclosan, diclofenac and carbamazepine affects embryonic development of medaka fish (*Oryzias latipes*). Chemosphere.

[CR17] Oliveira R, Domingues I, Koppe Grisolia C, Soares AM (2009). Effects of triclosan on zebrafish early-life stages and adults. Environ Sci Pollut Res Int.

[CR18] Orsi M, Noro MG, Essex JW (2011). Dual-resolution molecular dynamics simulation of antimicrobials in biomembranes. J R Soc Interface.

[CR19] Pai VB, Nahata MC (2000). Cardiotoxicity of chemotherapeutic agents: incidence, treatment and prevention. Drug Saf.

[CR20] Pycke BF, Geer LA, Dalloul M, Abulafia O, Jenck AM, Halden RU (2014). Human fetal exposure to triclosan and triclocarban in an urban population from Brooklyn, New York. Environ Sci Technol.

[CR21] Saley A, Hess M, Miller K, Howard D, King-Heiden TC (2016). Cardiac toxicity of triclosan in developing zebrafish. Zebrafish.

[CR22] Sandborgh-Englund G, Adolfsson-Erici M, Odham G, Ekstrand J (2006). Pharmacokinetics of triclosan following oral ingestion in humans. J Toxicol Environ Health A.

[CR23] Shim J, Weatherly LM, Luc RH, Dorman MT, Neilson A, Ng R, Kim CH, Millard PJ, Gosse JA (2016). Triclosan is a mitochondrial uncoupler in live zebrafish. J Appl Toxicol.

[CR24] Shirsath N, Rathos M, Chaudhari U, Sivaramakrishnan H, Joshi K (2013). Potentiation of anticancer effect of valproic acid, an antiepileptic agent with histone deacetylase inhibitory activity, by the cyclin-dependent kinase inhibitor P276-00 in human non-small-cell lung cancer cell lines. Lung Cancer.

[CR25] Stummann TC, Hareng L, Bremer S (2008). Embryotoxicity hazard assessment of cadmium and arsenic compounds using embryonic stem cells. Toxicology.

[CR26] Tafurt-Cardona Y, Suares-Rocha P, Fernandes TC, Marin-Morales MA (2015). Cytotoxic and genotoxic effects of two hair dyes used in the formulation of black color. Food Chem Toxicol.

[CR27] Waldmann T, Rempel E, Balmer NV, Konig A, Kolde R, Gaspar JA, Henry M, Hescheler J, Sachinidis A, Rahnenfuhrer J, Hengstler JG, Leist M (2014). Design principles of concentration-dependent transcriptome deviations in drug-exposed differentiating stem cells. Chem Res Toxicol.

[CR28] Weatherly LM, Shim J, Hashmi HN, Kennedy RH, Hess ST, Gosse JA (2016). Antimicrobial agent triclosan is a proton ionophore uncoupler of mitochondria in living rat and human mast cells and in primary human keratinocytes. J Appl Toxicol.

[CR29] Wei L, Qiao P, Shi Y, Ruan Y, Yin J, Wu Q, Shao B (2017). Triclosan/triclocarban levels in maternal and umbilical blood samples and their association with fetal malformation. Clin Chim Acta.

