# Supplementary figures and images for: Functional cardiotoxicity assessment of cosmetic compounds using human-induced pluripotent stem cell-derived cardiomyocytes
Source: Arch Toxicol. 2017 Sep 22;92(1):371–81. doi: 10.1007/s00204-017-2065-z (PMC5773645; doi:10.1007/s00204-017-2065-z)

Normalized cell index

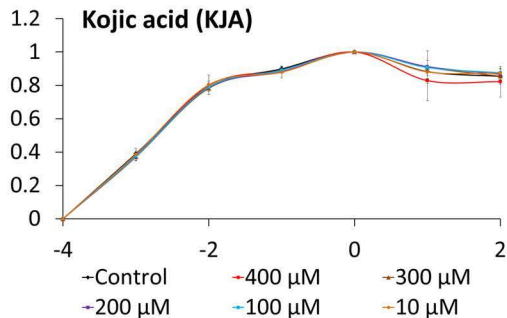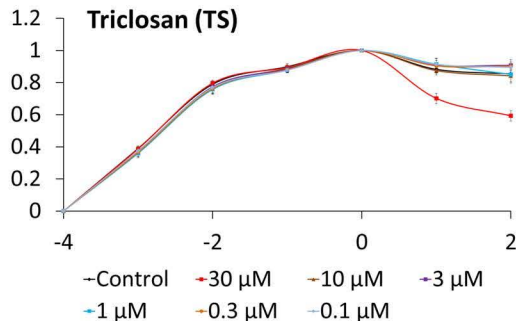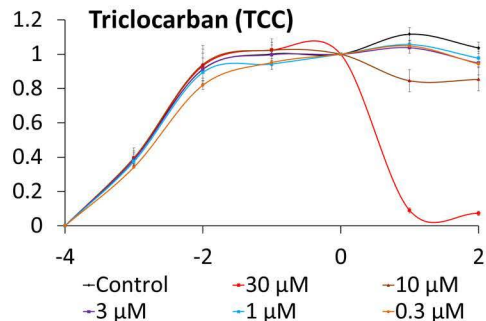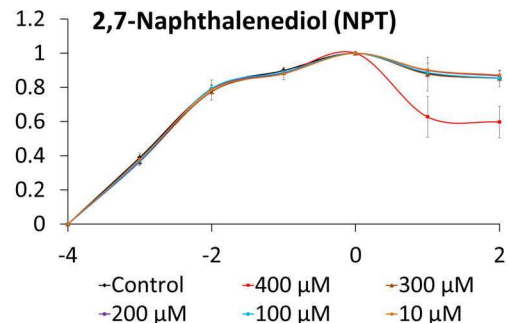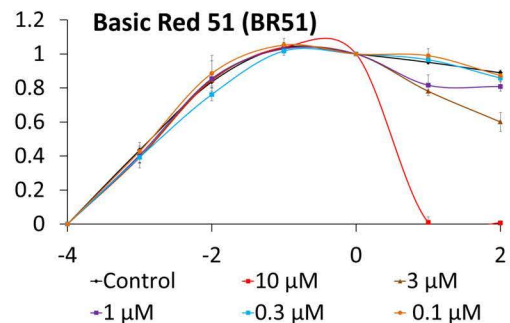

Time in days

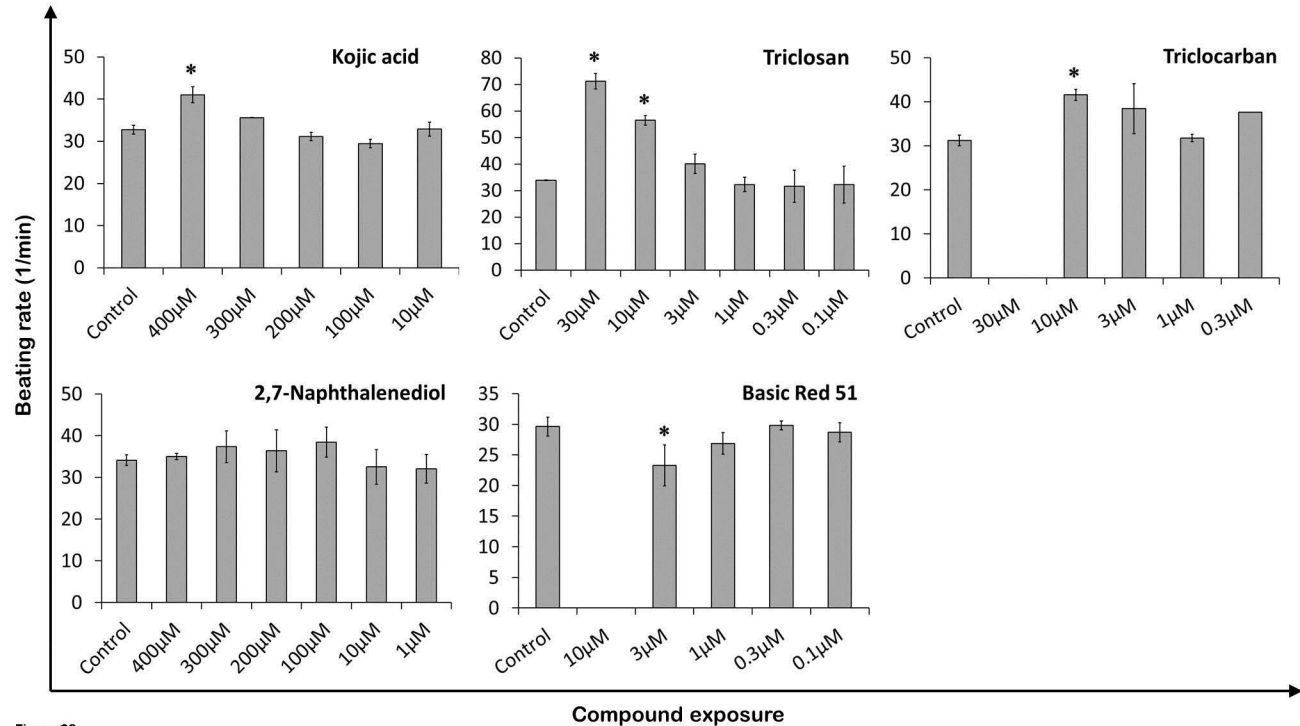

Figure S2

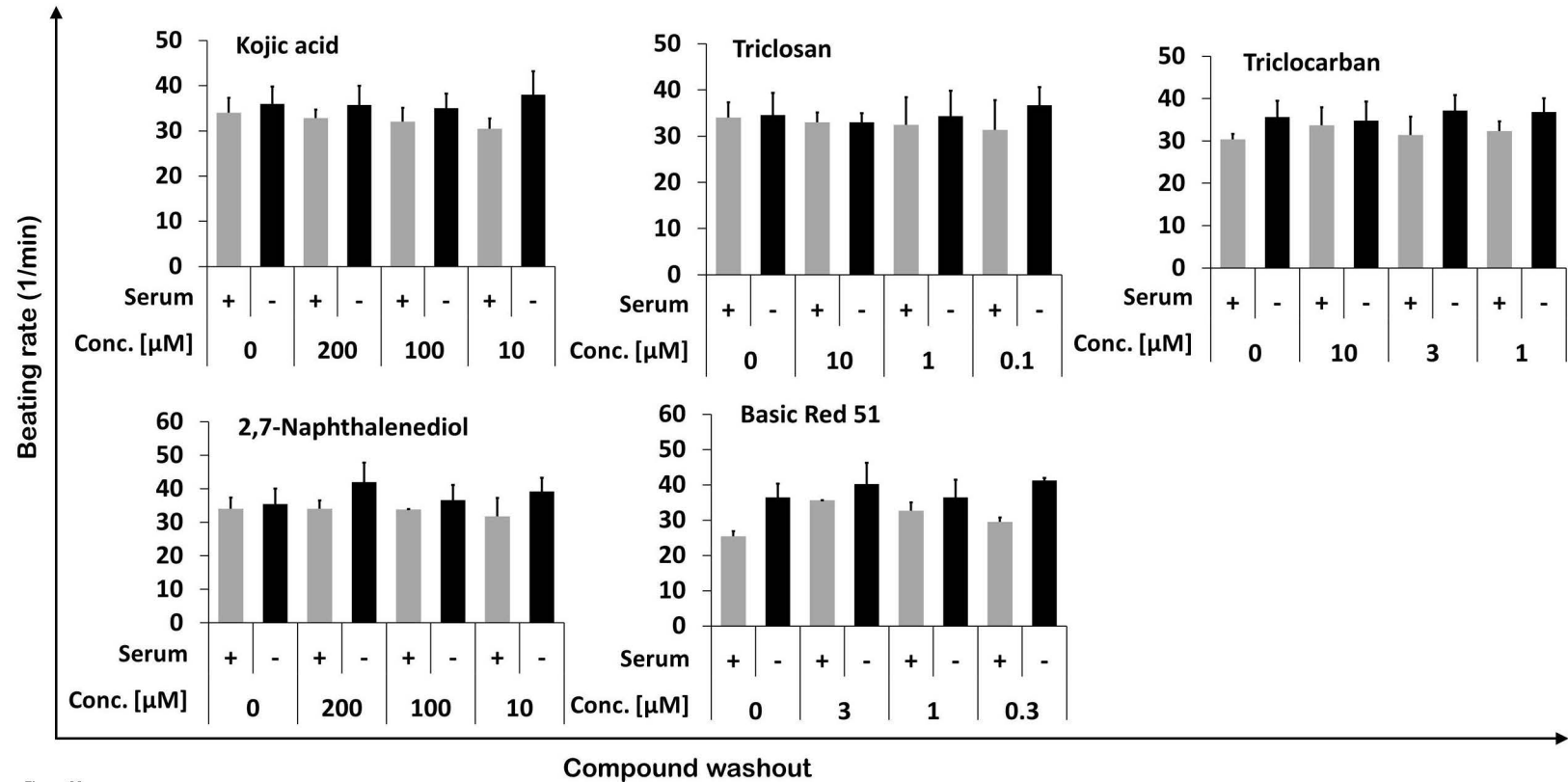

Figure S3

Supplement: Supplementary file 1 — Supplementary Fig. S1 Impedance-based cytotoxicity assessment of cosmetic test compounds in hiPSC-CMs in the presence of serum. Cytotoxic effect of test compounds in hiPSC-CMs was determined after 2 days of exposure using the xCELLigence RTCA Cardio software version 1.0. CI were normalized on day 0 just before start of compound exposure. Data represent mean ± SD, n = 3. Supplementary Fig. S2 Determination of arrhythmogenic effect of cosmetic test compounds on the beating rate of hiPSC-CMs in the presence of serum. Beating rate data were collected after 2 days of test compound exposure using the xCELLigence RTCA Cardio software version 1.0 at threshold 12. Numerical data presented as mean ± SD (n = 3), *p < 0.05 for the compound exposed vs control hiPSC-CMs. Supplementary Fig. S3 Evaluation of adverse effect of test compounds on the beating rate of hiPSC-CMs in the presence and absence of serum after 48 h compound washout. Beating rate data were obtained from the xCELLigence RTCA Cardio software version 1. In bar graph, error bar indicates mean ± SD (n = 3) (PDF 398 kb) [file 204_2017_2065_MOESM1_ESM.pdf]

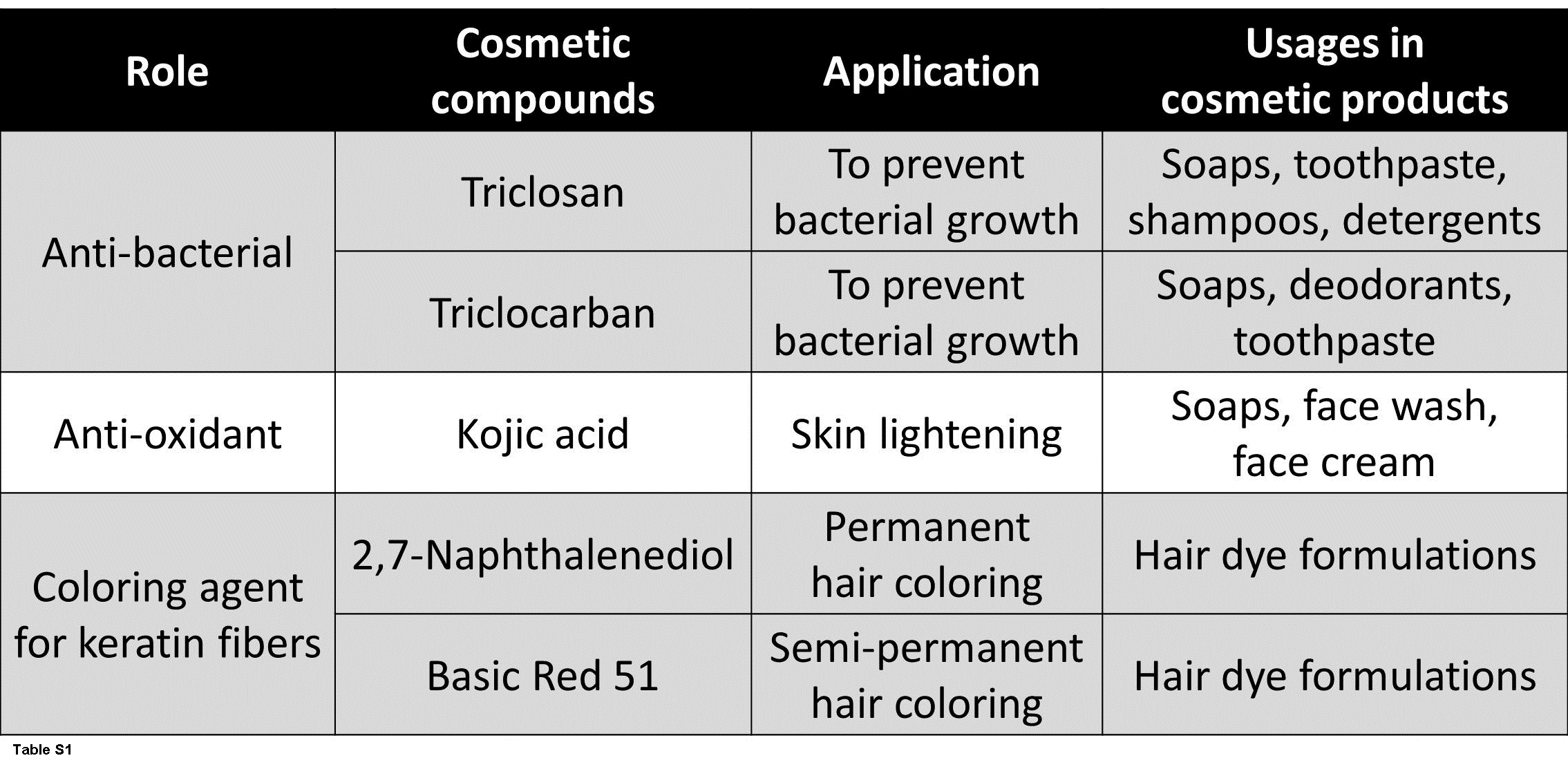

Supplement: Supplementary file 2 — Supplementary table S1. Classification and applicability of the cosmetic ingredients in commercial cosmetic products (TIFF 875 kb) [file 204_2017_2065_MOESM2_ESM.tiff]
